# Gooey stuff: the psychophysics of unpleasantness in response to touching liquids

**DOI:** 10.1098/rspb.2025.2244

**Published:** 2025-11-26

**Authors:** Müge Cavdan, Maja Fehlberg, Roland Bennewitz, Knut Drewing

**Affiliations:** ^1^ Justus Liebig University Giessen, 35390 Giessen, Germany; ^2^ Center for Mind, Brain, and Behavior (CMBB), Marburg 35032, Germany; ^3^ INM—Leibniz Institute for New Materials, 66123 Saarbrücken, Germany; ^4^ Department of Physics, Saarland University, 66123 Saarbrücken, Germany

**Keywords:** haptic perception, unpleasantness, viscosity, liquidity, material perception

## Abstract

There is a growing scientific interest in material unpleasantness, yet the role of distinct physical parameters in perceptual and affective haptic experiences with liquids remains to be fully understood. To address this, we investigated how perceptual qualities of liquids relate to measurable physical properties and unpleasantness during active touch. We prepared 15 custom liquid samples using everyday materials. Rheological measurements showed that samples varied between physical viscosity 1mPa⋅s and 45Pa⋅s. Participants explored each sample using circular rubbing motions with their index fingers. A camera system tracked finger movements, and a force sensor revealed applied normal forces, pull-off force (PoF) and the coefficient of friction (CoF). We compared these physical properties with the perceptual dimensions from our earlier work: perceived viscosity and slipperiness. Perceived viscosity correlated strongly with both physical viscosity and PoF, but not with CoF. Conversely, perceived slipperiness was associated with CoF, but not PoF or physical viscosity, demonstrating distinct links between physics and perception of liquids. Interestingly, PoF but not CoF was significantly linked to unpleasantness, suggesting that PoF but not CoF is crucial for liquid unpleasantness. These findings advance our understanding of how distinct physical properties relate to perceptual and affective experiences of liquids.

## 1. Introduction

We rely on our haptic sense to navigate and make sense of the world around us. From the moment we wash our face in the morning, we are continuously evaluating the properties of everything we interact with: e.g., the temperature of the water, the texture of the towel and the pressure of our hands on our skin. These subtle haptic cues guide our decisions: Is the water too cold? Is the towel soft enough? Am I pressing too hard? Even in such a routine act, haptics plays a crucial role in shaping our experience. It not only allows us to interpret the sensory properties of materials and objects but also evokes affective reactions [1]— for instance, softness of the towel might evoke feelings of comfort, while sudden coldness of water splashing against the face can trigger alertness. In fact, sensory and affective qualities are often closely linked. This relationship has been studied with a strong focus on solid materials (e.g., [2–7]). However, our everyday experiences also often involve fluids and semi-fluids such as lotions, gels and various food items. While some studies have focused on the sensory qualities of fluids, in particular the perception of viscosity [8–12], the present study goes beyond that by focusing on how viscosity, friction and pull-off force (PoF) relate to both sensory and affective liquid qualities.

For solid materials, pleasantness is associated with specific sensory qualities, including roughness [2,3,5], temperature [13,14] and softness [4,7,15]. Pioneering work by Major [4] demonstrated as early as 1895 that soft and smooth textures tend to feel pleasant, whereas stiff and rough surfaces feel unpleasant. These findings have been corroborated by others employing both physical and perceptual measures. Pleasantness and perceived softness have been shown to correlate positively with object compliance [7], while perceived roughness and unpleasantness increased with greater inter-element spacing of textures [3].

For liquids, the topic of pleasantness has received comparatively little attention. Nevertheless, a few studies have investigated their perceptual qualities based on physically well defined samples [8–10,16]. One earlier study [8] examined a broad range of Newtonian silicone fluids — fluids whose resistance to flow remains constant irrespective of the applied shear rate — with viscosities ranging from 10.3 to 95000 mPa·s. In a magnitude estimation experiment, participants evaluated perceived viscosity by shaking, stirring blindfolded or stirring with vision. Across the three exploration styles, perceived viscosity increased with physical viscosity following a power-law relationship. Interestingly, a more recent work demonstrated that humans reliably discriminate viscosities only above approximately 1800 mPa·s, when stirring liquids with a finger or a spatula [10]. Although the results of these two studies may seem at odds, the differences likely stem from the different tasks and their implementation: while the magnitude estimation reflects how strong a stimulus is felt on average, the discrimination task assesses noticeable differences using a limited range of stimulus differences, thereby focusing on reliable discrimination.

The perception of liquids has also been explored in applied domains like cosmetic science of skincare products (see [17]). In these settings, how a product feels (e.g., soft, sticky, etc.) is often examined through a combination of physical measurements with data from trained individuals (e.g., [18–20]). For instance, the frictional properties of cosmetic foundations have been measured using a tribometer to mimic the interaction that occurs when human fingers rub together during application [21]. Notably, correlations were found between the frictional signals and the sensory qualities *smoothness*, *silky feeling*, *velvety feeling*, *softness* and *skin-adhesion*, which were evaluated by three experts. Similar relationships have been reported between sensory qualities like spreadability and cohesiveness and a combination of rheological and texture analysis measurements [22–24]. Although such studies in cosmetic science often employ standardized industry protocols and expert participants to obtain precise measurements, they frequently fall short of capturing the natural movements and perceptual dynamics that occur during everyday interactions.

How liquids are perceived has been studied in perception and cosmetic science, yet which feelings they evoke have received much less attention. Guest *et al.* [25] investigated both the perceptual and affective dimensions of liquids. They found four sensory (*lubricating*, *textured*, *silken* and *viscous*) and two affective (*comfortable* and *arousing*) factors based on experiences of liquids on the volar forearm or underarm. Moreover, the study examined how different liquid stimuli influenced arousal and comfort across these two body regions without establishing concrete connections between perceived and affective qualities of liquids. Recently, we investigated how perceived viscosity relates to unpleasantness during finger exploration [26]. Our results revealed that the dimensions *viscosity* and *slipperiness* represented our liquid samples. Importantly, only perceived viscosity, not slipperiness, was related to unpleasantness. Nonetheless, this prior work focused solely on perceptual responses and did not incorporate physical measurements of either finger dynamics or material properties. Overall, although previous work on liquids has addressed the perceptual and physical aspects of viscosity, the connection between these properties and affective responses such as pleasantness has yet to be systematically examined in the context of fingertip exploration.

To address this gap, here we focus on how sensory and affective qualities of liquids relate to physical measures of viscosity, friction, and PoF. In an active haptic exploration experiment, we characterized the physical attributes of friction and PoF, i.e. the amount of force required to separate the finger’s skin from a contact surface [27], while individuals explored liquid samples of varied viscosities, quantified with rheological measurements. To examine the relationship between physical properties and sensory qualities, we correlated the physical measurements with perceptual ratings from [26]. By combining real fingertip measures with naive participants’ affective and perceptual judgments, our approach offers novel insights into the connection between measurable physical properties and subjective haptic experience. As this study bridges psychophysics and materials science, we provide methodological details that may exceed the conventions of either field individually to make the work accessible across disciplines.

## 2. Methods

### (a) Participants

Power analysis was conducted to ascertain the required sample size, based on a strong correlation (*p* = 0.7), 80% power, and alpha error probability of 0.05 [28]). The sample size for conducting the correlation analysis was 11. Accordingly, 11 healthy volunteers—including 2 of the authors—participated in the experiment (age range = 21–33, $M_{\text{age}}$= 28.6, *SE* = 1.04, 5 males). The participation of the authors was limited to the physical measurements (e.g., PoF). An extra analysis without the authors’ data did not change conclusions in any way. The study was ethically approved by the local ethics committee at Justus Liebig University Giessen (approval number: 2022-003) and performed following the Helsinki Declaration without preregistration [29]. Participants gave written informed consent for their participation.

### (b) Viscous stimuli

We used 15 different samples with varying viscosity. Everyday materials honey and hand cream were used as a base and were mixed with water, glycerine or starch. For each mixture, 10 g of base material was combined with either 3 or 6 g of one of the mixing materials (e.g., 10 g of honey + 3 g of starch, 10 g of honey + 6 g of starch). This resulted in a total of 12 mixtures (2 base materials × 3 mixing materials × 2 amounts). Additionally, the base materials were also used in their pure form (without any added materials), and water was used as a control material (see electronic supplementary material, table S1 for the recipes).

### (c) Force and position measurements

**Figure 1.**
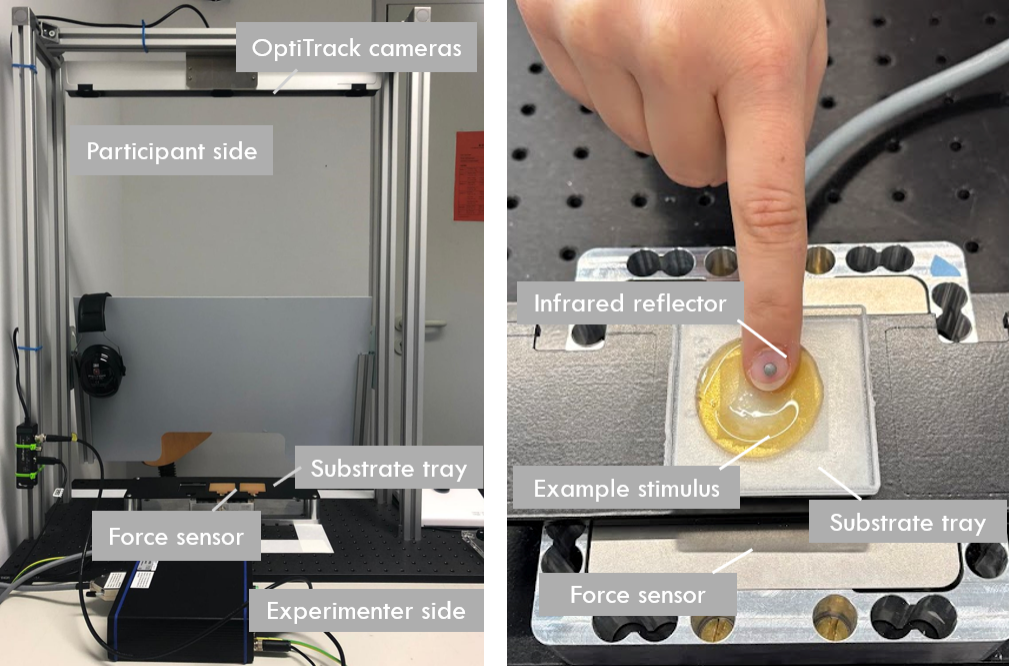
Experimental setup for measuring physical contact properties. Left: overview of the setup, illustrating the participant side, experimenter side, OptiTrack cameras for motion tracking, force sensor and substrate tray. Right: close-up of a participant's finger on an example stimulus placed in the substrate tray. The infrared reflector attached to the fingernail enables tracking, while the force sensor beneath the tray records applied forces.

To capture both motion and force data, an infrared-light-reflective marker tracked by a camera system (OptiTrack, V120: Trio) was used to capture the fingernail’s position during active exploration, while a 3-axis force sensor (ME-Messsysteme, K3D120, 50 N) simultaneously measured interaction forces exerted during exploration (figure 1). Both systems were synchronized and sampled at a frequency of 120 Hz. The sliding speed of the finger was determined as the distance travelled per time step (i.e., 8.33 ms).

To analyse the physical interaction between the fingertip and the sample, a quantity of 1.5–2.2 g of the samples was placed in a sandblasted polystyrene tray measuring 5 × 5 cm with a 5 mm high edge to prevent spillage. The surface had an average roughness ($S_{\text{a}}$) of 14.875 µm and a root mean square slope ($S_{\text{dq}}$) of 1.176. The tray was placed on the force sensor, which recorded the forces exerted by participants while exploring the sample. The fingertip friction force $\left(F_{\text{F}}\right)$ was calculated using data points where the normal force $\left(F_{\text{N}}\right)$ exceeded 0.1 N, indicating contact. The friction force was determined as


$$F_{\text{F}}=\sqrt{F_{x}^{2}+F_{y}^{2}},$$

where $F_{x}$ and $F_{y}$ are the lateral forces in the *x*- and *y*-directions. The coefficient of friction (CoF) was calculated as $F_{\text{F}}/F_{\text{N}}$ and median values are reported for each trial, i.e. for each exploration of one sample by one participant.

At the end of their haptic exploration of a sample, participants lifted their fingers, pulling them out of contact with a surface. The PoF was defined as the absolute value of the maximum negative normal force recorded during this action (similar approach to [27]). PoF values were normalized by dividing each value by the participant’s mean PoF.

### (d) Rheology

The viscosity η of the samples was determined by measuring the rate-dependent shear stress required to shear the liquids between a base plate and a parallel rotating plate. These shear flow curves were recorded at a temperature of 28°C, which is a typical temperature at the human fingertip [30]. The parallel-plate geometry had a diameter of 25 mm and a gap height of 0.5 mm (Anton-Paar MCR302e rheometer). Shear rates were logarithmically increased from 10 to 1000 ${\text{s}}^{-1}$.

To estimate the relevant range of shear rates for analysing fingertip friction results, we re-enacted the experiment described in [26] using the honey sample. The typical exploration procedure involved shearing the sample between the index finger and thumb (rubbing for viscous materials in [31,32] similar to circular motion in [33]). The thickness of the spread honey film was then measured by optical coherence tomography (OCT, Thorlabs Ganymede OCT-SD). The shear rate was subsequently estimated by dividing the sliding speed by the thickness of the liquid sample film.

### (e) Procedure

Participants explored each sample once (figure 1). Before starting the experiment and before each trial on a new sample, participants washed their hands. A waiting period of 10 min followed each hand-washing to allow fingertip moisture to be restored naturally. During the exploration phase, participants used their straight right index finger to perform circular motions [27] on the sample surface, completing at least five circles. After the exploration, they lifted the finger from the sample. Following each measurement, the sample was removed from the tray and replaced with a new one. This was repeated until the exploration procedure was completed for every sample.

### (f) Earlier experiment: unpleasantness and perceptual qualities

To examine the relationship between perceptual and physical measures, we drew on unpleasantness and perceptual judgments collected in our earlier work using the same set of samples. In this previously reported viscosity and unpleasantness experiment [26], 19 volunteers had participated ($M_{\text{age}}$ = 25.7, age range = 19–39, *SE* = 1.29), who were recruited through university circular emails. None of the authors participated in this earlier perceptual experiment.

In this previously published experiment, in two blocks, individuals had rated the unpleasantness and perceptual qualities of each sample. During the experiment, a curtain blocked visual access to the stimuli, earplugs and active-noise cancelling headphones (Sennheiser HD 4.50) reduced any sounds generated due to exploration, and a box restricted possible odours from the stimuli. Together, these measures ensured that ratings were solely based on the haptic exploration experience.

In the first block, upon exploring each substance without any time or movement restrictions with their right (i.e., dominant) hand, individuals rated how unpleasant the sample was (1: very pleasant, to 7: very unpleasant). There was no definition for unpleasantness or any other contextual framing; participants were simply asked to indicate how unpleasant the stimulus felt to touch. After each trial, their hands were cleaned with the help of the experimenter. The experiment consisted of 45 trials (15 stimuli × 3 repetitions) with a randomized order of samples. In the second block, the participants again explored the samples as in the first block but this time rated how much different perceptual qualities apply to each sample (1: not at all, to 7: very). These qualities were sticky, semi-fluid, cold, slippery, warm and viscous (*klebrig*, *zaehfluessig*, *kalt*, *rutschig*, *warm* and *viskoes*, respectively, in German). Adjectives were presented on the monitor screen in a random order upon exploration. After all the adjectives were rated, the experimenter helped the participants clean their hands. This block consisted of 15 trials — one for each sample. The experiment took approximately 75 min, including welcome, instructions, and cleaning.

### (g) Data analysis

Normalized CoF values were calculated for each trial by dividing the CoF value by each individual’s mean CoF over all samples.

In our earlier experiment, a covariance-based principal component analysis (PCA) was conducted on individual ratings, and component scores for each sample were extracted using the Bartlett method [34]. We used the covariance-based approach considering all perceptual ratings were on the same scale, and our goal was to preserve the natural variance structure of the data for component extraction. To test the relationship between physical properties and perceived qualities of samples, the average Bartlett scores obtained from each sample and dimension (i.e. viscosity and slipperiness from [26]) were correlated with the logarithm of the average CoF, the logarithm of physical viscosity, and the logarithm of PoF values. All physical property values (physical viscosity, CoF and PoF) were thereafter presented in logarithmic form.

We additionally tested the relationship between unpleasantness and physical sample properties using the average unpleasantness scores per sample. In each subsection, *p*-values for the correlation analyses are Bonferroni-corrected for multiple testing.

Here, we focus on the relationship between perceptual and affective responses and physical measurements, including CoF and PoF, which are averaged across participants. In an accompanying physics paper [35], we analysed individual-level data on friction, sliding speed and normal force from the same experiment, demonstrating a transition from full-film lubrication with high-viscosity samples to boundary lubrication with low-viscosity samples

## 3. Results

### (a) Rheology

The optical measurements by OCT revealed that the thickness of the spread honey film was 250 µm (electronic supplementary material, figure S3). No measurement for the film thickness was possible with the hand cream owing to its opaqueness. From the motion capture data, we calculated the median speed of haptic exploration for all participants. The measured exploration speeds ranged between 3.8 and 8.8 cm s^−1^, with a median speed of 5 cm s^−1^ across participants. A characteristic shear rate of 200 s^−1^ was estimated by dividing the median speed of 5 cm s^−1^ by the film thickness of 250 µm. For the presentation of viscosity results, we hereafter use the physical viscosity values for this characteristic shear rate. To account for varying shear rates in the complex flow under the sliding fingertip, we also consider viscosity at shear rates of 80 and 500 s${,}^{-1}$.

**Figure 2.**
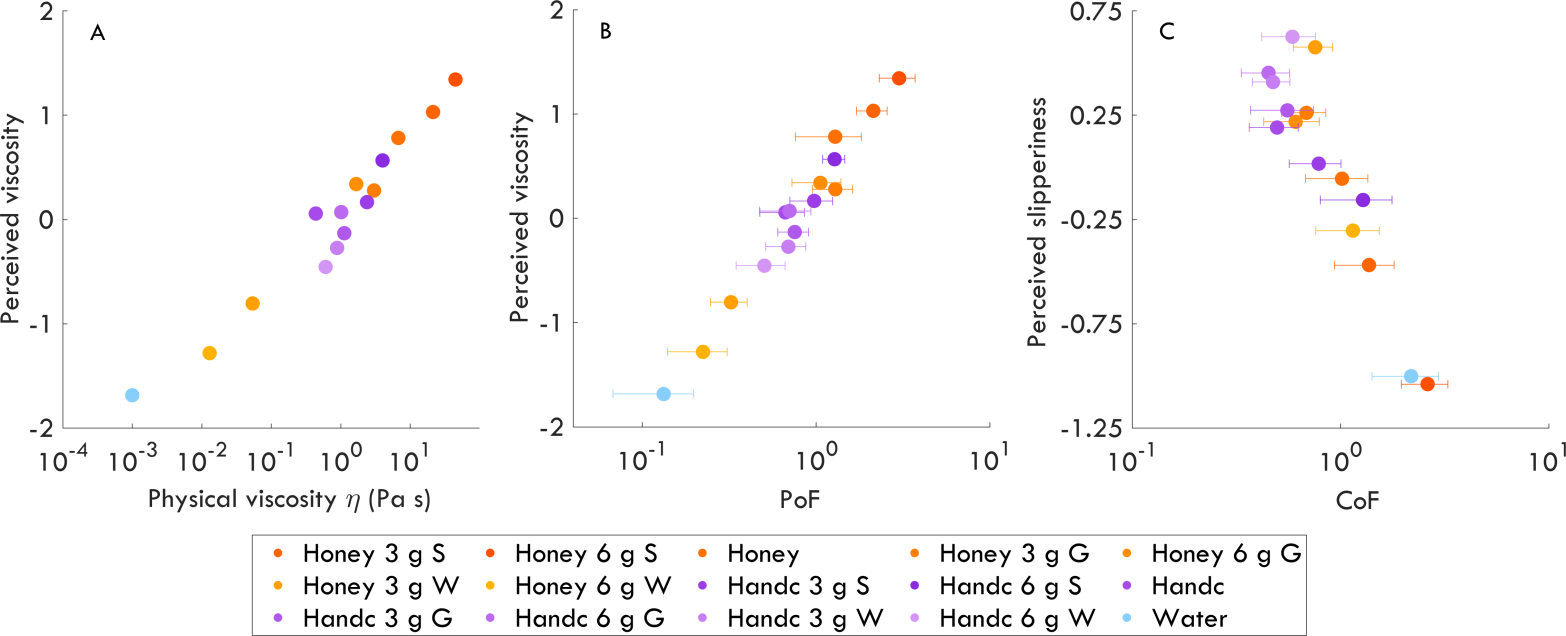
The relationships between perceived viscosity and physical viscosity (A), perceived viscosity and PoF (B), and perceived slipperiness and CoF (C). Orange colours represent honey, while purple colours represent hand cream (Handc) samples. Blue shows water (control sample). Error bars correspond to the standard deviation of the mean. S, starch; G, glycerine; W, water.

The rheology results confirmed that our samples had a wide range of physical viscosity between 1 mPa·s and 44.83 Pa·s. The results indicated that starch significantly increased physical viscosity in all samples, while water and glycerine reduced it for honey, but not for hand cream (figure 2). The non-Newtonian behaviour of hand cream exhibited shear-thinning in all handcream samples, as indicated by the decrease in viscosity with increasing shear rate, while for honey this was the case only with 3 and 6 g starch. In contrast, the viscosity of honey samples with water and glycerin mixtures remained constant with the increasing shear rate.

### (b) Perceived qualities of the samples

One of the primary goals of this study was to examine the relationship between the perceived qualities and physical properties of viscous samples. To facilitate the comparison, this section provides a brief overview of the results from the perception experiment. First, raw adjective ratings per sample and participant were submitted to a PCA. A Kaiser–Meyer–Olkin value of 0.64 indicated an adequate sampling rate [36], and Bartlett’s sphericity test ($\chi^{2}$(21) = 522.96, p<0.01) exhibited significant correlations.

Therefore, we extracted perceptual dimensions from a covariance-based PCA. The first rotated component explained 37.06% of the variance, with high loadings of the adjectives *semi-fluid*, *sticky*, *viscous*, *warm* and *cold*, and we called it viscosity. Notably, this component also encompassed a temperature-related dimension, as indicated by the opposite loadings of warm (positive) and cold (negative) [26]. The second rotated component accounted for 24.32% of the variance and had high loadings of *slick* and *slippery*, which we called slipperiness. We extracted the dimension scores using Bartlett’s method (i.e., scores for the viscosity and slipperiness dimensions) and averaged for each sample. These scores were used for the subsequent correlation analyses in the following sections. Conducting PCA on the adjective ratings allowed us to identify underlying perceptual dimensions while reducing redundancy among correlated descriptors. This approach provides a more parsimonious and interpretable framework for examining the relationship between perceptual judgments and physical material properties [37].

### (c) The relationships between perceptual qualities and physical sample properties

First, we tested whether perceived viscosity was correlated with physical viscosity, PoF and CoF. The relationship between perceived viscosity and physical viscosity was strong: *r* = 0.97, *p* < 0.001 (figure 2A). A similarly strong correlation was found with physical viscosity values for lower and higher shear rates, where the spread of data for non-Newtonian hand cream was slightly different (electronic supplementary material, figure S4). Similarly, the relationship between perceived viscosity and PoF was strong and statistically significant (r = 0.99, p<0.001; figure 2B), but the perceived viscosity did not show such a correlation with CoF (r = 0.10, p = 0.73; *p*-values are corrected for three tests; electronic supplementary material, figure S2A). Overall, perceived viscosity was significantly associated with physical viscosity and PoF but showed no clear relationship with CoF.

Additionally, we inspected whether perceived slipperiness was correlated with CoF (figure 2C), PoF (electronic supplementary material, figure S2B) and physical viscosity (electronic supplementary material, figure S2C). Perceived slipperiness was negatively correlated with CoF (r = −0.93, p<0.001). However, the relationships between perceived slipperiness and PoF and perceived slipperiness and physical viscosity were not statistically significant: r = −0.09, p = 0.75; r = 0.04, p = 0.89, respectively (*p*-values are corrected for three tests). These findings indicate that perceived slipperiness is strongly associated with CoF, but show no such indication with PoF or physical viscosity.

### (d) The relationship between qualities of liquid samples and unpleasantness

**Figure 3.**
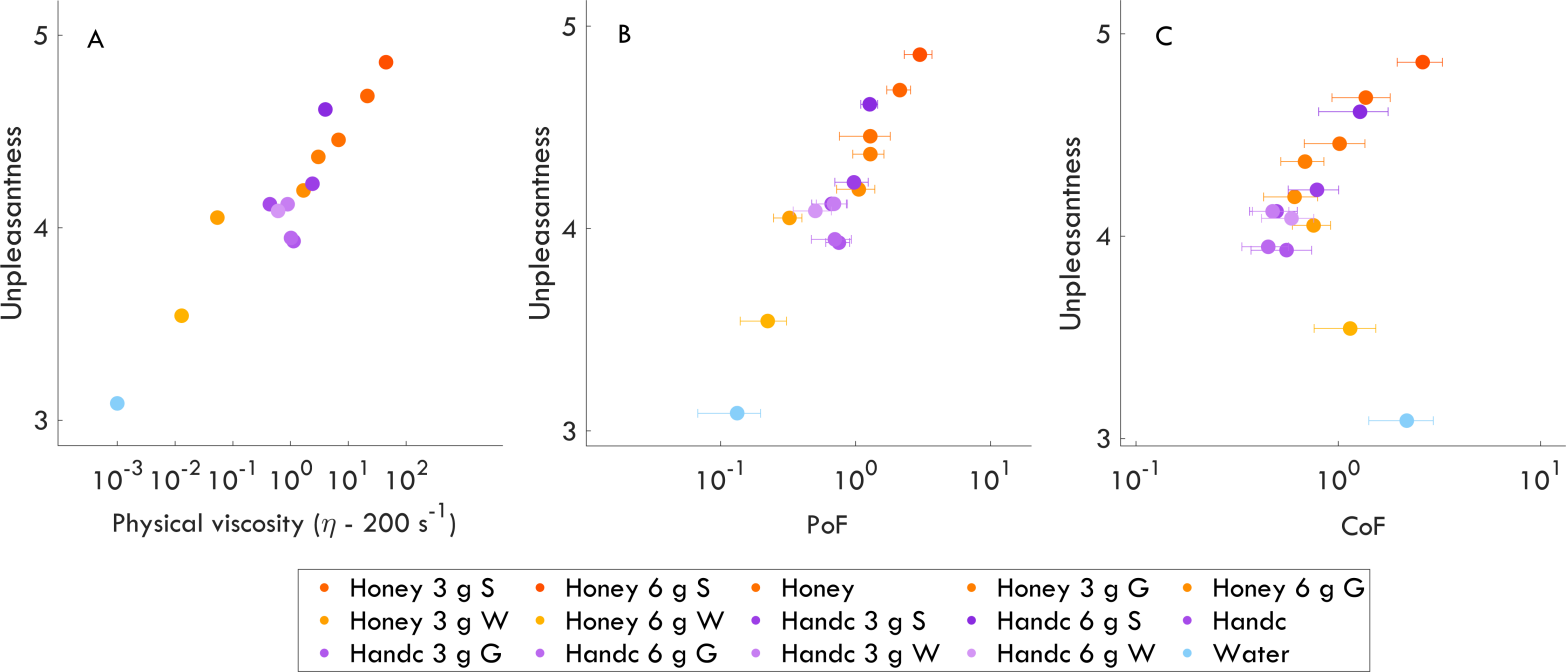
The relationships between unpleasantness and physical sample qualities: physical viscosity (A), PoF (B) and CoF (C), respectively. Orange colours represent honey while purple colours represent hand cream (Handc) samples. Blue shows water (control sample). Error bars correspond to the standard deviation of the mean. S, starch; G, glycerine, W, water.

Another main goal of the current work was to investigate the relationship between the physical properties of liquid samples and unpleasantness. Specifically, we tested whether unpleasantness was correlated with physical parameters (figure 3). While unpleasantness was strongly correlated with physical viscosity (r=0.94, p<0.001; similar for viscosity at different shear rates; see electronic supplementary material, figure S4) and PoF (r=0.94, p<0.001), it was not significantly correlated with CoF (r=0.10, p=0.73; *p*-values are corrected for three tests). Thus, unpleasantness appears to be strongly related to both physical viscosity and PoF but not CoF. Notably, unpleasantness in our population was consistently well understood, as reflected by the high agreement in ratings across individuals (see fig. 3 in [26]).

### (e) Pull-off force and fingertip friction

**Figure 4.**
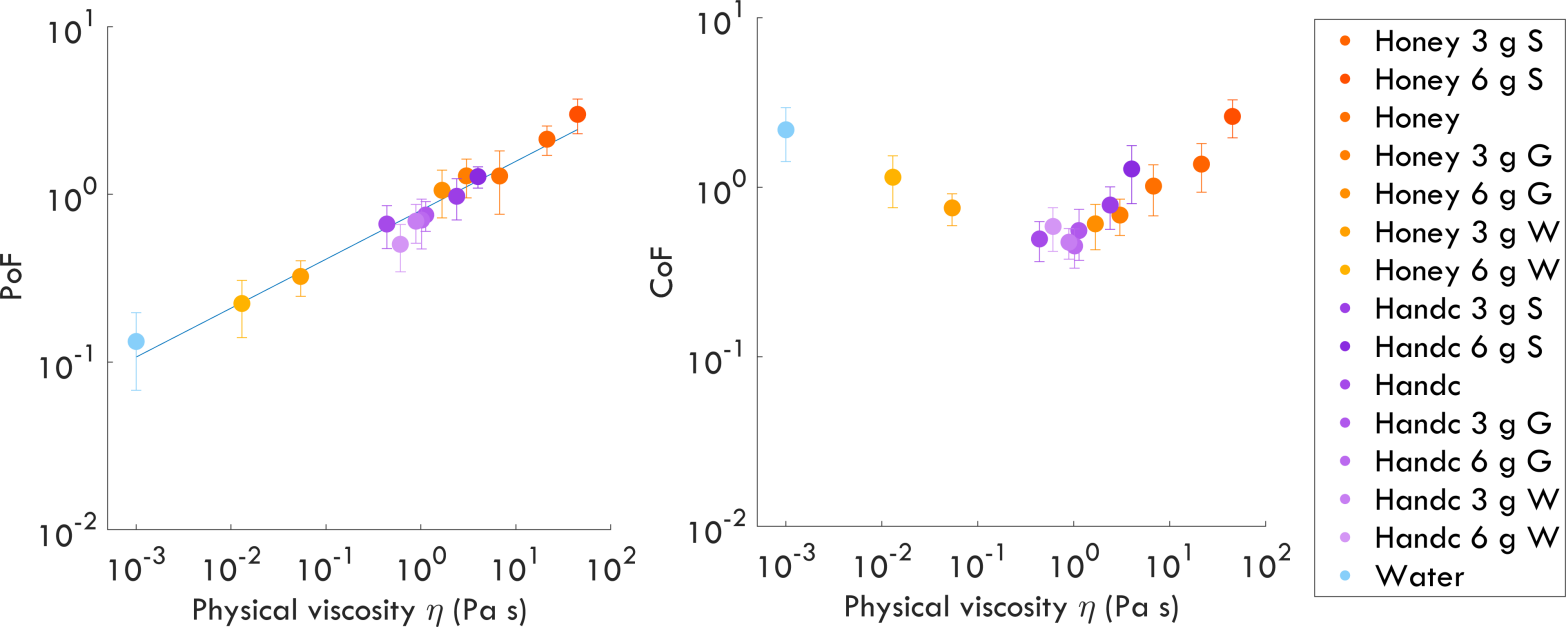
Left: mean normalized pull-off force (PoF) required to detach the finger from the samples as a function of their viscosity (*η*). The average normalized PoF follows a power-law relationship with *η* at a shear rate $\dot{\gamma }$ = 200 s^−1^, with an exponent of 0.32 ± 0.02. Right: mean coefficient of friction (CoF) as a function of physical viscosity across the samples. S, starch; G, glycerine; W, water; Handc, hand cream.

The PoF — the force required to lift the fingertip off the samples—increased with the physical viscosity of the sample (*r* = 0.98, *p* < 0.001). Theoretically, PoF is expected to scale with $\sqrt{\eta }$, where η represents the sample’s physical viscosity. However, PoF may also be influenced by pulling velocity and the film thickness prior to detachment [38]. The complex flow in the liquid upon fingertip retraction may be governed not only by the shear viscosity quantified in this study. The stretching and breaking of an elongated liquid neck between the substrate and fingertip would instead be controlled by surface tension and extensional viscosity [39]. However, the average pull-off length, i.e., the position at which the maximum PoF is detected, was only 1 mm, with the largest pull-off length in any trial being 5.2 mm (electronic supplementary material, figure S5). These values are smaller than the typical contact radius, and the maximum PoF is reached before the liquid is stretched into an extensional neck. We therefore assume that contributions of extensional viscosity to this early stage of pull-off are small. Our results indicate that physical shear viscosity is a strong predictor of the normalized PoF across individuals (figure 4, left). Our results indicate that physical viscosity is a strong predictor of the normalized PoF across individuals (figure 4, left). However, the relationship between CoF and physical viscosity (figure 4, right) was not statistically significant (*r* = −0.03, *p* = 0.92).

Fingertip friction coefficients depend on the lubrication provided by the viscous sample films. Key parameters influencing this lubrication are the physical viscosity $\dot{\gamma }$, the sliding speed v and the applied normal force $F_{N}$, which together determine the thickness of the film at the contact between the sliding fingertip and surface. We observed that participants tended to use lower sliding speeds (r = −0.66, p=0.008) and higher normal forces (r = 0.78, p=0.001) when interacting with samples of higher physical viscosity. Across samples, the variations were typically within 20% of the average sliding speed and within 50% of the average normal force (see electronic supplementary material, figure S1).

## 4. Discussion

Liquid substances are part of our daily routines—for instance, when using liquid soap or spreading sunscreen on the skin at the beach. In the current study, we explored the relationship between unpleasantness, perceptual qualities and potential physical indicators, including the CoF, PoF and physical viscosity. To this end, we created 15 samples from everyday materials, systematically varying their physical properties, which were verified through rheological measurements. A PCA from perceptual data [26] revealed that the same samples varied along two independent perceptual dimensions: viscosity and slipperiness. These perceptual dimensions were to compared against the measured physical properties. Perceived viscosity was strongly correlated with physical viscosity and PoF, but not CoF. However, perceived slipperiness was correlated with CoF, but not PoF or physical viscosity. Importantly, when comparing unpleasantness with the physical measurements, we found strong relationships between unpleasantness and both physical viscosity and PoF, whereas no significant relationship was observed with CoF. These results highlight that CoF and PoF reflect distinct physical mechanisms underlying separate dimensions of haptic perception—slipperiness and viscosity—and their associated affective responses.

Our findings confirmed that the samples not only were perceived to vary in viscosity [26] but also exhibited differences in physically measured viscosity, which ranged from 1 mPa·s and 44.83 Pa·s. These results highlight that careful material selection and formulation — even using everyday substances — can yield physically well controlled liquid samples suitable for psychophysical research. Specifically, we found that adding starch to the base samples increased the physical viscosity of all samples, and water and glycerine reduced it only in honey (figure 2A).

Physical and perceived viscosity were strongly correlated, suggesting that individuals could perceive our liquid samples. Perceived viscosity exhibited an almost perfect linear correlation with the logarithm of physical viscosity, providing evidence that viscosity perception may follow Weber’s Law. Earlier work suggested that humans could only discriminate between viscous samples above *ca* 2 Pa·s [10,40], yet in our study, participants were able to provide systematic ratings even for samples falling well below this level. Moreover, the same perceptual trend was observed for samples above 2 Pa·s, showing that the findings hold across the full viscosity range we tested. These findings, therefore, question the relevance of this threshold for bare-finger free exploration conditions. This discrepancy could stem from differences in task demands — rating versus discrimination — or from exploration method variations used across studies. However, this should be interpreted with caution, given the differences in the stimulus characteristics and exploration method across studies. Our stimuli were non-Newtonian and therefore exhibited shear-dependent viscosity responses that are not directly comparable to the Newtonian fluids used in previous studies. Additionally, while the participants in [10] stirred the samples using a spatula or their fingers, our participants engaged in unrestricted, naturalistic exploration, which likely introduced different frictional cues. This distinction is also important, considering recent work has shown that individuals not only use stirring [32] but also rubbing and pulling ([31] stretching material between the fingers) to assess viscosity. Limiting participants to a single hand movement may, therefore, restrict the perceptual information they could obtain. An intriguing future path would be to expand the range of viscosities and investigate whether participants could reliably discriminate between lower and higher viscosities through free exploration.

Interestingly, both physical and perceived viscosity were strongly associated with PoF but not CoF, while perceived slipperiness was closely linked to CoF alone. The dissociation suggests that PoF and CoF act as distinct *physical correlates* of two separable perceptual dimensions—viscosity and slipperiness. Specifically, it suggests that viscosity and slipperiness might emerge from distinct physical cues—PoF conveying information about material resistance (or internal flow), and CoF reflecting surface interactions during glide. This link between PoF and viscosity aligns with previous work identifying a relationship between PoF and perceived stickiness (or glide) [27] — one of the defining qualities within our viscosity dimension. The similarly high correlations of both perceived viscosity and unpleasantness with PoF and physical viscosity likely arise from the strong interdependence between PoF and physical viscosity itself (figure 4, left). We propose that the initial phase of pulling the finger off the liquid surface up to the maximum PoF, where the distance is still smaller than the contact radius, is dominated by shear flow [38], and that this shear-dominated mechanism underlies the tight coupling of PoF and physical shear viscosity, thereby influencing both perception of viscosity and unpleasantness. In many trials, participants pulled a thin neck out of the liquid, which extended up to a couple of centimetres for more viscous samples. However, the forces in this late phase are lower than the maximum PoF, which is shown here to correlate to the measured shear viscosity.

Relatedly, unpleasantness was strongly correlated with PoF and physical viscosity but not CoF. Specifically, as unpleasantness increased, so did the physical viscosity and PoF. This resonates with a previous study in the cosmetic industry, where increased stickiness was shown to negatively impact consumer acceptance of a product [20]. A similar finding was also observed in oral–tactile research, where the viscosity of food was positively correlated with unpleasantness [41,42]. Conversely, perceived slipperiness, which was correlated with CoF, was not significantly related to unpleasantness. This is interesting considering instinct theory [43], which would predict a repulsion impulse occurring upon skin contact with slippery and slimy substances. One possible explanation is that our study exclusively provided haptic information while restricting other sensory information. When these other senses are limited or absent, the typical aversive response to slippery substances (e.g., visual [44]) may be attenuated. Taken together, these findings highlight that PoF and perceived (also physical) viscosity play important roles in determining how unpleasant a liquid feels. In contrast, slipperiness does not appear to be a consistent factor in unpleasantness across the wide range of viscosities, where two distinct lubrication regimes cause a non-monotonic friction response.

Although we determined the sample size through power analysis and it was sufficient to detect the expected effects, the relatively small number of participants (*n* = 11) may constrain the generalizability of our findings to broader populations (e.g., older adults). Future research with larger and more diverse samples could further validate and extend these conclusions.

Previous research has consistently shown that, in the context of solid surfaces and materials, a higher CoF is associated with increased unpleasantness [6,45,46]. However, our findings reveal an interesting divergence from this pattern in the case of liquid samples, where friction did not show a consistent correlation with unpleasantness. This discrepancy may reflect different interaction mechanics involved in liquid versus solid contexts. While exploring fluids, frictional cues are shaped by dynamic flow and changing skin–material interactions, which may alter their affective responses. As a result, the relationship between friction and affective response may follow a multi-regime pattern, unlike the more monotonic associations observed with solid materials. Future work could further clarify whether such different regimes exist by systematically varying liquid properties to identify how they modulate haptic unpleasantness.

## 5. Conclusion

In conclusion, our findings highlight how the physical properties of fluids relate to how (un)pleasant these materials feel. Specifically, unpleasantness appears to be closely tied to the force required to separate the skin from a contact surface, rather than how slippery the substance is. Beyond advancing our understanding of the sensory and affective processing of non-solid substances, this research also has practical implications for designing comfortable haptic experiences in everyday products.

## Data Availability

Data from this study are available with open access at https://osf.io/wzvqp/overview?view_only=4df6779bc55c4dcd8cbd3a2300b55a94. Supplementary material is available online [47].
